# “Role of kidney function and concentrations of BAFF, sPD-L1 and sCD25 on mortality in hospitalized patients with COVID-19”

**DOI:** 10.1186/s12882-022-02924-2

**Published:** 2022-09-02

**Authors:** Ladan Mansouri, Senka Sendic, Sebastian Havervall, Charlotte Thålin, Stefan H. Jacobson, Joachim Lundahl

**Affiliations:** 1 Department of Clinical Science and Education, Karolinska Institutet, Södersjukhuset, Stockholm, Sweden; 2 Division of Nephrology, Department of Clinical Sciences, Karolinska Institutet, Danderyd University Hospital, Stockholm, Sweden; 3 Division of Internal Medicine, Department of Clinical Sciences, Karolinska Institutet, Danderyd University Hospital, Stockholm, Sweden

**Keywords:** COVID-19, BAFF, sCD25, SPD-L1, Kidney function

## Abstract

**Background:**

Chronic kidney disease (CKD) is a recognized risk factor for severe complications in COVID-19. Our objective was to analyze the association between kidney function / T and B lymphocyte modulatory factors and risk of mortality in COVID-19 patients.

**Methods:**

In-hospital and 30‐day mortality were analyzed in COVID‐19 patients (*n* = 110). Plasma levels of selected T and B cell modulators were analyzed and correlated to mortality risk. A subgroup of sex- and eGFR-matched COVID-19 patients was compared to CKD patients without infection and healthy subjects.

**Results:**

COVID-19 patients who died in hospital and within 30 days had significantly higher BAFF and sCD25 plasma levels than survivors. In logistic regression models patients with high BAFF, sCD25 and sPD-L1 levels had significantly higher risk of both in-hospital and 30-day mortality while there was no association to eGFR. In the subgroup analysis, a higher level of BAFF, IFN-α, sCD25, sPD-L1 and a lower level of sCD40L was observed in COVID-19 patients compared to the CKD group with corresponding kidney function.

**Conclusions:**

We demonstrate that kidney function and concentrations of BAFF, sCD25 and PD-L1, independent of previously recognized risk factors; age, male gender, and leukocytosis are associated with risk of in-hospital and 30-day mortality in patients with COVID-19. These data indicate the significance of adaptive immune system modulators in COVID-19 and motivate further analysis to identify new potential prognostic and therapeutic approaches.

**Supplementary Information:**

The online version contains supplementary material available at 10.1186/s12882-022-02924-2.

## Background

Presence of chronic kidney disease (CKD) has recently emerged as one of the most common risk factors for severe Coronavirus disease (COVID)-19 [1]. Patients with advanced CKD (stages 4–5, estimated glomerular filtration rate (eGFR) < 30 ml/min/1.73m^2^), dialysis or a kidney transplant are among the groups with the highest risk of death from COVID-19, and notably even after adjustment for covariates. This risk is higher than the risk conferred by for example diabetes, asthma, and chronic heart disease [1–9]. Yet, the increased risk is also evident in moderate CKD (eGFR < 60 ml/min/1.73m^2^) and even a mild increase in serum creatinine seems to associate with increased risk of severe COVID-19 and mortality [3, 9, 10].

Impairment in the innate and adaptive immune system as well as disturbances in cellular immunity predisposes to an increased risk of severe infections, virus-associated cancers, and a weakened vaccine response in CKD [11–13]. However, an exaggerated response of the immune systems can also occur in CKD, resulting in increased production and decreased clearance of proinflammatory chemokines and cytokines, which in turn leads to systemic inflammation with sequelae [12].

Recent studies have suggested that not only antibodies, but also memory B cells, and specially T cells, have an important role in immunity towards COVID-19 [14, 15]. Vaccination is an important component to retard the Severe Acute Respiratory Syndrome Coronavirus (SARS-CoV)-2 pandemic, but vaccine protection in CKD may be less effective [11, 12]. The clinical and immunological factors that contribute to the greater vulnerability for severe COVID-19 in CKD, and a potential lower effect of vaccination have not fully been explored and new information may provide valuable insights into new therapeutic approaches.

Given a significant role of the adaptive immune system in the clinical course of COVID-19 and in the outcome of the vaccination program in patients with reduced kidney function, we analyzed selected factors that are involved in B cell function; B cell activating factors (BAFF), Interleukin (IL-4), Interferon (IFN)-α and T cell function: soluble Programmed death-ligand (sPD-L)-1, soluble Cluster of differentiation (sCD)-25, sCD40L, sCD95, and their relation to in-hospital and 30-day mortality in hospitalized patients with COVID-19. Moreover, to expand the knowledge of the impact of reduced kidney function in COVID-19 patients, we included a CKD group that matched a subgroup of COVID-19 patients for sex and eGFR in parallel with a sex- and age-matched healthy control group.

We hypothesized that these inflammatory molecules are affected in COVID-19 patients and are associated to the mortality risk. We also tested the hypothesis that the levels of these denominators were not solely a consequence of reduced kidney function in COVID-19 patients.

## Methods

### Study population

One hundred ten COVID-19 patients admitted to Danderyd University Hospital, Stockholm, Sweden were included between April 9 and June 8, 2020. Patients were admitted to hospital at different stages and at different time points of their COVID-19 disease, depending on the different clinical signs and symptoms. Clinical characteristics in a subgroup of these patients have been presented elsewhere [16]. Inclusion criteria were age > 18 years, diagnosis of COVID-19 with reverse‐transcriptase polymerase chain reaction (RT‐PCR) viral RNA detection of nasopharyngeal or oropharyngeal swabs or clinical presentation, with known kidney function (eGFR). 33 sex- and eGFR-matched patients with mild to severe CKD and 35 healthy sex and age-matched subjects from the PROGRESS study [17] were included as controls (Table 1). The PROGRESS study was a cohort study wherein patients with CKD and matched healthy controls were systematically enrolled to be followed and analyzed under the course of five years. They had no ongoing infection at the time and the plasma samples from these individuals at time of recruitment was used in the current study as controls.

**Table 1:** Comparison between patients with COVID-19, patients with chronic kidney disease (CKD) and healthy subjects. The comparison between groups was analyzed by nonparametric Kruskal–Wallis test and a *P* < 0.05 was considered a significant difference

	COVID-19 (*N* = 110)		CKD patients (*N* = 33)		Healthy subjects (*N* = 35)		*P (K-W)*
	Median	IQR 25–75%	Median	IQR 25–75%	Median	IQR 25–75%	
Age (years)	60	50–69	55	44.5–58	50	39–57	< 0.001^a^
BMI^1^ (kg/m^2^)	28	24.6–31.6	25.0	23.5–28.0	24	22–27	< 0.001^b^
Creatinine (µmol/l)	73	57.5–89	122	107–160	72	66–77	< 0.001^c^
eGFR^2^ (ml/min/1.73m^2^)	84	67–90	53	40–65	102	96–108	< 0.001^d^
CRP^3^ (mg/l)	99	63–174	1.6	0.9–4.3	0.89	0.31–2.2	< 0.001^e^
*B cell modulation*							
BAFF^4^ (pg/ml)	824	576–1099	448	395–551	403	365–449	< 0.001^f^
IL-4^5^ (pg/ml)	104	88–124	134	111–208	119	98–156	< 0.001^ g^
IFN-α^6^ (pg/ml)	18.5	9–36.5	10	4.5–25	8	5–32.5	0.02^ h^
*T cell modulation*							
sCD25^7^/IL-2Rα^8^ (pg/ml)	1178	931–1587	734	582–808	363	309–441	< 0.001^i^
sCD40L (pg/ml)	1515	1220–1884	7663	5688–9955	7239	4985–9564	< 0.001^j^
sPD-L1^9^ (pg/ml)	146	96–227	83	61–102	39	30–48	< 0.001^ k^
sCD95/Fas (pg/ml)	6083	4727–7292	8519	7373–10,112	5572	4917–6058	< 0.001^ l^

^1^ Body mass index

^2^ Estimated glomerular filtration rate

^3^ C-reactive protein

^4^ B-cell activating factor

^5^ Interleukin 4

^6^ Interferon-α

^7^ Soluble cluster of differentiation 25

^8^ Interleukin 2 receptor- α

^9^ Soluble programmed death-ligand 1

^a^ *P* < 0.01 comparing COVID-19 and CKD patients and *P* < 0.0001 vs healthy controls, ^b^ *P* < 0.05 comparing COVID-19 and CKD patients and *P* < 0.0001 vs healthy controls, ^c^ *P* < 0.0001 comparing COVID-19 and CKD patients and NS vs healthy controls, ^d^ *P* < 0.0001 comparing COVID-19 and CKD patients and vs healthy controls, ^e^ *P* < 0.0001 comparing COVID-19 and CKD patients and *P* < 0.001 vs healthy controls, ^f^ *P* < 0.0001 comparing COVID-19 vs CKD patients and vs healthy controls, ^g^ *P* < 0.0001 comparing COVID-19 vs CKD patients and NS vs healthy controls, ^h^ *P* < 0.01 comparing COVID-19 and CKD patients and NS vs healthy controls, ^i^ *P* < 0.0001 comparing COVID-19 vs CKD patients and vs healthy controls, ^j^ *P* < 0.0001 comparing COVID-19 and CKD and vs healthy controls, ^k^ *P* < 0.0001 comparing COVID-19 and CKD patients and vs healthy controls, ^l^ *P* < 0.0001 comparing COVID-19 and CKD patients and NS vs healthy control

In-hospital mortality and mortality within 30 days from admission (30-day mortality) were obtained from medical records. The portion of patients who had treatment with low molecular weight heparin or glucocorticoids at the time of sampling was 78% and 5% respectively. High doses of steroids, antiviral medication or hydroxychloroquine were not clinically applied during the study period. 32% of the patients had treatment with either an angiotensin-converting-enzyme inhibitor or an angiotensin II receptor blocker. Most patients were hospitalized at a general ward (86%), while 10% of patients were admitted to an intermediate care unit and 4% of patients to the intensive care unit at the time of blood sampling.

### Demographic and laboratory data

Routine laboratory blood analyses (plasma creatinine, C-reactive protein (CRP), white blood cell (WBC) count, neutrophil count, and lymphocyte count in patients with COVID-19 were performed at the time of admission at the Karolinska University Hospital Laboratory, Stockholm, Sweden. We present both plasma creatinine and estimated glomerular filtration rate (eGFR, CKD-EPI formula) since COVID-19 patients may comprise both patients with CKD and patients with acute kidney injury (AKI), and eGFR may be more accurate in CKD than in AKI. COVID-19 patients had a median eGFR of 84 ml/min/1.73m^2^ which was chosen as a threshold value and the patients with eGFR below this threshold value were regarded having reduced kidney function. We analyzed neutrophil-to-lymphocyte ratio (NLR) in COVID-19 patients (median 4, IQR 2.4–8.0) since it has previously been shown that this ratio is higher in more severe COVID-19 disease and a predictive factor for mortality [18, 19].

### Analysis of circulatory inflammatory factors using multiplex immunoassay

EDTA tubes (Vacutainer, Becton Dickinson, UK) were applied to collect blood from patients after hospital admission and plasma was prepared by centrifugation at 2,000 × g for 20 min at room temperature and stored in -80 °C freezer. After thawing, the plasma samples were centrifuged at 16,000 × g for 4 min and diluted 1:2 according to the manufacturer’s instructions. We analyzed the levels of the BAFF, IL-4, sCD25/IL-2Rα, sCD40L, sPD-L1, sCD95/Fas and IFN-α, using the Human Magnetic Luminex Assay premixed 21-plex (Catalog nr. LXSAHM-21, Lot nr. L134758, R&D systems, Minnesota**,** USA). The plates were analyzed by Bio-plex MAGPIX Multiplex reader (Bio Rad, California, USA). A pooled plasma was used for inter-plate comparisons as control.

### Statistical analysis

Patients with COVID-19 (*n* = 110) and patients with CKD (disease controls) (*n* = 33) and healthy controls (*n* = 35) were compared. We also compared laboratory results in 29 CKD patients (of 33 disease controls) who were matched for kidney function with the subgroup of COVID-19 patients with reduced eGFR (eGFR ≤ 84 ml/min/1.73m^2^) as explained earlier. Comparisons between three groups were performed using the nonparametric Kruskal–Wallis test, and comparison between two groups with Mann–Whitney U-test, due to non-normal distribution of results. We applied the Dunn’s post hoc test for multiple comparisons following the Kruskal–Wallis test. Scatter plots were prepared in GraphPad Prism 8.3 (GraphPad Software, Inc., USA) in which whiskers represent 25–75% interquartile range (IQR) and the median is shown by a line. GraphPad Prism 8.3 (GraphPad Software, Inc., USA), STATISTICA version 10 (StatSoft, Inc., USA) and IBM SPSS Statistics 25 (IBM Corp., USA) were applied for statistical analysis. Spearman´s rank correlation test was used to determine the relationship between eGFR and demographic and laboratory data, as well as between cytokines and neutrophil-to-lymphocyte ratio. Chi-square test was applied to analyze differences between stratified groups. Logistic multiple regression analyses were applied for analysis of mortality risk. Due to the low number of patients, we were able to adjust analyses for only one factor at a time. As age has been shown to associate with mortality in several other observational studies, we primarily adjusted our analyses for age, as well as sex and eGFR.

## Results

### Comparisons of COVID-19 patients with CKD patients and healthy subjects

Table 1 displays the demographic characteristics and laboratory findings of all the participants. In terms of B cell modulation, the concentrations of BAFF and IFN-α were significantly higher and IL-4 lower in the entire patient group with COVID-19 (*n* = 110) compared to CKD patients (Table 1). Concerning T cell modulation, sCD25 and sPD-L1 levels were significantly higher, while sCD40L and sCD95 levels were significantly lower in COVID-19 patients compared to both control groups. Demographic and routine laboratory data as well as concentrations of immune modulators in patients with COVID-19, patients with CKD and healthy subjects are shown in Table 1.

Since correlation between neutrophil-to-lymphocyte ratio and BAFF levels in Covid-19 has been reported [20], we analyzed this relationship in our patient group, which was significantly correlated (*r* = 0.26, *P* = 0.01). Considering the role of IFNs in production of BAFF in immune cells [21] as well as synergy of BAFF and IL-4 in B cell physiology [22], we analyzed the relationship between IFN-α and BAFF and BAFF and IL-4 (Appendix Fig. 1).

The correlation between sCD25 and sPD-L1 levels, the T cell activator and immune checkpoint, in COVID-19 patients, was significant (*r* = 0.37, *P* < 0.0001) as well as the relationship between sCD25 and sCD95 (*r* = 0.46, *P* < 0.0001) (Appendix Fig. 1).

### Immune modulators in relation to kidney function in patients with Covid-19

Correlations by Spearman’s rank test between inflammatory modulators and eGFR are presented in Table 2. There were significant negative correlations between eGFR and neutrophil-to-lymphocyte ratio, BAFF, IL-4, sCD25, sPD-L1 and sCD95 in patients with Covid-19.

**Table 2:** Correlation (Spearman´s rank correlation, Spearman’s rho) between eGFR and laboratory findings

	Spearman’s rho
WBC^a^ count (× 10^9^/l)	-0.105
Neutrophil count (× 10^9^/l)	-0.098
Lymphocyte count (× 10^9^/l)	0.368*
NLR^b^	-0.279*
*B cell modulation*	
BAFF^c^ (pg/ml)	-0.356*
IL-4^d^ (pg/ml)	-0.311*
IFN-α^e^ (pg/ml)	-0.055
*T cell modulation*	
sCD25^f^/IL-2Rα^g^ (pg/ml)	-0.488*
sCD40L (pg/ml)	-0.058
sPD-L1^h^ (pg/ml)	-0.445*
sCD95/Fas (pg/ml)	-0.430*

^a^ White blood cells

^b^ Neutrophil-leukocyte ratio

^c^ B-cell activating factor

^d^ Interleukin 4

^e^ Interferon-α

^f^ Soluble cluster of differentiation 25

^g^ Interleukin 2 receptor- α

^h^ Soluble programmed death-ligand 1

Statistical significance: ^*^P < 0.01

### Comparisons of COVID-19 patients (eGFR ≤ 84 ml/min/1.73m^2^) with matched CKD patients

As mentioned in methods, 29 out of 33 CKD patients had eGFR below the median of ≤ 84 ml/min/1.73m^2^ and they were compared with a sex and eGFR-matched subgroup of COVID-19 patients with reduced kidney function (*n* = 29). Analysis showed a higher level of BAFF, IFN-α, sCD25, sPD-L1 and a lower level of sCD40L in COVID-19 patients compared to CKD patients with corresponding eGFR. The differences in IL-4 and sCD95/Fas levels which we observed when comparing the larger groups of patients with COVID-19 (*n* = 110) and CKD (*n* = 33), were not observed in this subgroup analysis. Appendix Table 1 displays the differences between the two groups.

### In-hospital mortality and 30-day mortality in patients with COVID-19

Patients who died in-hospital were more often men with higher age (*P* < 0.05) and deceased patients had significantly higher CRP, WBC count and neutrophil-to-lymphocyte ratio and lower eGFR compared to patients who survived hospital stay (Table 3). Concentrations of BAFF and sCD25 were significantly higher in patients who died during hospital stay with a statistical trend also for sPD-L1 and sCD95.

**Table 3:** Characteristics of patients with COVID-19 who died (*n* = 15) in hospital or survived (*n* = 95)

**In-hospital outcome**						*P* (Chi-square analysis)
		Survived		Deceased		
		Count	%	Count	%	
Age (years)	Low^k^	53	56	3	20	= 0.01
(median)	High^l^	42	44	12	80	
Creatinine (µmol/l) (median)	Low	56	59	3	20	< 0.01
	High	39	41	12	80	
eGFR^a^ (ml/min/1.73m^2^) median	Low	44	46	12	80	< 0.05
	High	51	54	3	20	
WBC^b^ count (x10^i^/l) (median)	Low	52	55	2	13	< 0.01
	High	43	45	13	87	
Neutrophil count (x10^i^/l) (median)	Low	46	48	3	20	< 0.05
	High	49	52	12	80	
Lymphocyte count (x10^i^/l) (median)	Low	42	44	9	60	NS
	High	53	56	6	40	
NLR^c^ (median)	Low	46	48	2	13	= 0.01
	High	49	52	13	87	
CRP^d^ (mg/l) (median)	Low	51	54	3	20	< 0.05
	High	44	46	12	80	
*B cell modulation*						
BAFF^e^ (pg/ml) (median)	Low	52	55	3	20	= 0.01
	High	43	45	12	80	
IL-4^f^ (pg/ml) (median)	Low	50	53	5	33	NS
	High	45	47	10	67	
IFN- α^g^ (pg/ml) (median)	Low	50	53	4	27	0.06
	High	45	47	11	73	
*T cell modulation*						
sCD25^h^/IL-2Rα^i^ (pg/ml) (median)	Low	53	56	2	13	< 0.01
	High	42	44	13	87	
sCD40L (pg/ml) (median)	Low	48	51	7	47	NS
	High	47	49	8	53	
sPD-L1^j^ (pg/ml) (median)	Low	51	54	4	27	0.052
	High	44	46	11	73	
sCD95/Fas (pg/ml) (median)	Low	51	54	4	27	0.052
	High	44	46	11	73	

^a^ Estimated glomerular filtration rate

^b^ White blood cells

^c^ Neutrophil-leukocyte ratio

^d^ C-reactive protein

^e^ B-cell activating factor

^f^ Interleukin 4

^g^ Interferon-α

^h^ Soluble cluster of differentiation 25

^i^ Interleukin 2 receptor- α

^j^ Soluble programmed death-ligand 1

^k^ Low: lower than median. ^l^High: higher than median

When evaluating 30-day mortality, patients who died were mainly men (*P* < 0.05) and deceased patients had higher CRP, higher WBC count, lower lymphocyte count, higher neutrophil-to-lymphocyte ratio, higher BAFF, sCD25 and INF-α levels than patients who survived for 30 days (Table 4). A statistical trend towards higher concentrations for sPD-L1 and sCD95 was also observed in patients who died within 30 days.

**Table 4:** Characteristics of patients with COVID-19 who died (*n* = 12) within 30 days and patients who survived (*n* = 98) for 30 days

**30-day outcome**						*P* (Chi-square analysis)
		Survived		Deceased		
		Count	%	Count	%	
Age (years)	Low^k^	53	54	3	25	0.057
(median)	High^l^	45	46	9	75	
Creatinine (µmol/l) (median)	Low	56	57	3	25	< 0.05
	High	42	43	9	75	
eGFR^a^ (ml/min/1.73m^2^) median	Low	47	48	9	75	0.077
	High	51	52	3	25	
WBC^b^ count (x10^i^/l) (median)	Low	53	54	1	8	< 0.01
	High	45	46	11	92	
Neutrophil count (x10^i^/l) (median)	Low	48	49	1	8	< 0.01
	High	50	51	11	92	
Lymphocyte count (x10^i^/l) (median)	Low	42	43	9	75	< 0.05
	High	56	57	3	25	
NLR^c^ (median)	Low	48	49	0	0	= 0.001
	High	50	51	12	100	
CRP^d^ (mg/l) (median)	Low	53	54	1	8	< 0.01
	High	45	46	11	92	
*B cell modulation*						
BAFF^e^ (pg/ml) (median)	Low	53	54	2	17	< 0.05
	High	45	46	10	83	
IL-4^f^ (pg/ml) (median)	Low	51	52	4	33	NS
	High	47	48	8	67	
IFN-α^g^ (pg/ml) (median)	Low	52	53	2	17	< 0.05
	High	46	47	10	83	
*T cell modulation*						
sCD25^h^/IL-2Rα^i^ (pg/ml) (median)	Low	53	54	2	17	< 0.05
	High	45	46	10	83	
sCD40L (pg/ml) (median)	Low	51	52	4	33	NS
	High	47	48	8	67	
sPD-L1^j^ (pg/ml) (median)	Low	52	53	3	25	0.067
	High	46	47	9	75	
sCD95/Fas (pg/ml) (median)	Low	52	53	3	25	0.067
	High	46	47	9	75	

^a^ Estimated glomerular filtration rate

^b^ White blood cells

^c^ Neutrophil-leukocyte ratio

^d^ C-reactive protein

^e^ B-cell activating factor

^f^ Interleukin 4

^g^ Interferon-α

^h^ Soluble cluster of differentiation 25

^i^ Interleukin 2 receptor- α

^j^ Soluble programmed death-ligand 1

^k^ Low: lower than median. ^l^ High: higher than median

### Unadjusted, age-adjusted, sex-adjusted, and eGFR-adjusted risk of in-hospital mortality in patients with COVID-19

In unadjusted logistic regression analysis of risk of in-hospital mortality, age (*P* < 0.01, OR 1.071, CI 1.023–1.122), eGFR, CRP, WBC count, neutrophil count, neutrophil-to-lymphocyte ratio, BAFF, sCD25 and PDL-1 levels were significantly associated with risk of in-hospital mortality.

Risk of in-hospital mortality in patients with COVID-19, adjusted for age, is shown in Table 5. Patients with high CRP, WBC count, neutrophil-to-lymphocyte ratio and sCD25 had significantly higher risk of in-hospital mortality, with a statistical trend for BAFF. No association to eGFR was observed in the age adjusted model.

**Table 5:** Age-adjusted, Sex-adjusted and eGFR-adjusted logistic regression analysis, odds ratio (OR) with 95% confidence intervals (CI), of in-hospital mortality in patients with COVID-19

	**Age-adjusted**			**Sex-adjusted**			**eGFR-adjusted**		
	*P*-value	OR	95% CI	*P*-value	OR	95% CI	*P*-value	OR	95% CI
s-Creatinine (µmol/l)	NS	1.001	0.996–1.007	NS	1.001	0.995–1.007	-	-	-
eGFR^a^ (ml/min/1.73m^2^)	NS	0.983	0.955–1.012	= 0.010	0.969	0.946–0.993	-	-	-
CRP^b^ (mg/l)	= 0.01	1.008	1.002–1.015	= 0.010	1.008	1.002–1.015	= 0.01	1.008	1.002–1.014
WBC^c^ count (x10^i^/l)	= 0.001	1.438	1.167–1.771	= 0.001	1.341	1.136–1.582	< 0.01	1.266	1.080–1.484
Neutrophil count (x10^i^/l)	< 0.001	1.512	1.189–1.921	= 0.001	1.423	1.161–1.745	= 0.001	1.464	1.171–1.832
Lymphocyte count (x10^i^/l)	NS	1.127	0.342–3.715	NS	0.746	0.251–2.213	NS	1.163	0.375–3.613
NLR^d^	< 0.05	1.117	1.009–1.237	< 0.05	1.119	1.019–1.230	0.058	1.111	0.996–1.239
*B cell modulation*									
BAFF^e^ (pg/ml)	0.058	1.001	1.000–1.002	< 0.05	1.001	1.000–1.002	NS	1.001	1.000–1.001
IL-4^f^ (pg/ml)	NS	1.004	0.995–1.014	NS	1.002	0.994–1.009	NS	1.001	0.992–1.010
IFN- α^g^ (pg/ml)	NS	1.004	0.995–1.013	NS	1.002	0.994–1.010	NS	1.005	0.999–1.010
*T cell modulation*									
sCD25^h^/IL-2Rα^i^ (pg/ml)	< 0.05	1.000	1.000–1.001	< 0.05	1.000	1.000–1.001	NS	1.000	1.000–1.001
sCD40L (pg/ml)	NS	1.000	0.999–1.001	NS	1.000	0.999–1.001	NS	1.000	0.999–1.001
sPD-L1^j^ (pg/ml)	NS	1.003	0.999–1.008	< 0.05	1.004	1.000–1.009	NS	1.002	0.997–1.007
sCD95 (pg/ml)	NS	1.000	1.000–1.000	NS	1.000	1.000–1.000	NS	1.000	1.000–1.000

^a^ Estimated glomerular filtration rate

^b^ C-reactive protein

^c^ White blood cells

^d^ Neutrophil-leukocyte ratio

^e^ B-cell activating factor

^f^ Interleukin 4

^g^ Interferon-α

^h^ Soluble cluster of differentiation 25

^i^ Interleukin 2 receptor- α

^j^ Soluble programmed death-ligand 1

An analysis of in-hospital mortality adjusted for sex is shown in Table 5, in which concentrations of BAFF (*P* < 0.05), sCD25 (*P* < 0.05) and sPD-L1 (*P* < 0.05) were significantly associated with mortality. An analysis of in-hospital mortality adjusted for eGFR, showed no cytokine level associated with mortality (Table 5).

### Unadjusted, age-adjusted, sex-adjusted, and eGFR-adjusted risk of 30-day mortality in patients with COVID-19

In unadjusted logistic regression analysis of 30-day mortality the following parameters were significantly associated with risk of mortality: age (*P* < 0.05, OR 1.052, CI 1.004–1.102), eGFR, CRP, WBC count, neutrophil count, neutrophil-to-lymphocyte ratio, BAFF, sCD25 and sPD-L1.

Variables associated with 30-day mortality with COVID-19, adjusted for age, are shown in Table 6. Patients with high CRP, WBC count, neutrophil count, neutrophil-to-lymphocyte ratio, BAFF, sCD25 and sPD-L1 had significantly higher risk of 30-day mortality, with no significant association with eGFR. The same analysis adjusted for sex, showed a similar pattern (Table 6), while analysis adjusted for eGFR showed that high BAFF levels associated with increased risk of mortality (*P* < 0.05), (Table 6).

**Table 6:** Age-adjusted, Sex-adjusted and eGFR-adjusted logistic regression analysis, odds ratio (OR) with 95% confidence intervals (CI), of 30-day mortality in patients with COVID-19

	**Age-adjusted**			**Sex-adjusted**			**eGFR-adjusted**		
	*P*-value	OR	95% CI	*P*-value	OR	95% CI	*P*-value	OR	95% CI
s-Creatinine (µmol/l)	NS	1.000	0.994–1.006	NS	1.001	0.995–1.007	-	-	-
eGFR^a^ (ml/min/1.73m^2^)	NS	0.983	0.955–1.012	= 0.010	0.969	0.946–0.993	-	-	-
CRP^b^ (mg/l)	= 0.001	1.012	1.004–1.019	< 0.01	1.011	1.004–1.019	= 0.001	1.011	1.004–1.019
WBC^c^ count (x10^i^/l)	< 0.001	1.435	1.174–1.755	< 0.001	1.464	1.190–1.800	= 0.001	1.331	1.122–1.579
Neutrophil count (x10^i^/l)	< 0.001	1.589	1.232–2.015	< 0.001	1.626	1.243–2.128	= 0.000	1.613	1.237–2.103
Lymphocyte count (x10^i^/l)	NS	0.498	0.126–1.964	NS	0.455	0.125–1.656	NS	0.562	0.146–2.157
NLR^d^	= 0.01	1.155	1.034–1.291	= 0.01	1.151	1.033–1.283	= 0.01	1.158	1.031–1.301
*B cell modulation*									
BAFF^e^ (pg/ml)	< 0.05	1.001	1.000–1.002	= 0.01	1.001	1.000–1.002	< 0.05	1.001	1.000–1.002
IL-4^f^ (pg/ml)	NS	1.005	0.996–1.014	NS	1.003	0.995–1.011	NS	1.003	0.995–1.011
IFN- α^g^ (pg/ml)	NS	1.005	0.997–1.014	NS	1.003	0.995–1.011	NS	1.004	0.995–1.012
*T cell modulation*									
sCD25^h^/IL-2Rα^i^ (pg/ml)	< 0.05	1.000	1.000–1.001	< 0.05	1.001	1.000–1.001	NS	1.000	1.000–1.001
sCD40L (pg/ml)	NS	1.000	0.999–1.001	NS	1.000	0.999–1.001	NS	1.000	1.000–1.001
sPD-L1^j^ (pg/ml)	< 0.05	1.005	1.000–1.009	< 0.05	1.005	1.001–1.010	NS	1.004	0.998–1.009
sCD95 (pg/ml)	NS	1.000	1.000–1.000	NS	1.000	1.000–1.000	NS	1.000	1.000–1.000

^a^ Estimated glomerular filtration rate

^b^ C-reactive protein

^c^ White blood cells

^d^ Neutrophil-leukocyte ratio

^e^ B-cell activating factor

^f^ Interleukin 4

^g^ Interferon-α

^h^ Soluble cluster of differentiation 25

^i^ Interleukin 2 receptor- α

^j^ Soluble programmed death-ligand 1

## Discussion

Our main finding in the present study is that kidney function and factors that are partly involved in B cell activation (BAFF), T cell activation (sCD25) and suppression (sPD-L1), apart from previously recognized risk factors; age, male gender, leukocytosis, and high neutrophil-to-lymphocyte ratio are associated with risk of mortality and add important predictive information in hospitalized patients with COVID-19.

In the present study we included a CKD group without COVID-19 infection that was sex- and eGFR-matched with a subgroup of COVID-19 patients with reduced kidney function together with a healthy control group. The rationale for this design was to evaluate whether patients with impaired kidney function during COVID-19, an acute infection, exhibit a cytokine signature different from that in matched CKD patients, with a chronic systemic inflammatory state, but without an acute COVID-19 infection. Indeed, the observed differences between the entire COVID-19 group and the control groups persisted when we compared the subgroup of COVID-19 patients with CKD patients with similar kidney function. This denotes that the immune signature that associates with mortality, is not exclusive for COVID-19 patients with kidney impairment and should be regarded as a risk feature regardless of kidney function. It is however important to mention some patients with COVID-19 may have had a concurrent episode of AKI which may have influenced the results*.* We report that the level of BAFF, a cytokine important for activation, development, and proper selection of B cells, associates with mortality in COVID-19. This finding is in-line with previous studies which have shown a positive correlation between the number of circulatory B cells and BAFF levels in COVID-19 patients [20] and that B cells do have a major role in SARS-CoV-2 infection due to their rapid production of neutralizing antibodies [23, 24]. However, it is noteworthy that BAFF is not only a major B cell modulator but also modifies other immune cells such as T cells [25].

The production of BAFF is associated with type I IFNs when viruses, including SARS-CoV-2, invade cells such as monocytes, macrophages, and dendritic cells [22, 25, 26] but there are heterogeneous IFN-α profiles reported in COVID-19 patients [26, 27]. Herein, we report a higher level of IFN-α compared to our two control groups but without any clear association with mortality.

Since disturbances in the T cell compartment characterize COVID-19 patients [28] and different subpopulations are proposed to predict the clinical course of the disease [29, 30], we focused on two factors related to T cell function in our study, namely sCD25/ IL-2Rα (activation) and sPD-L1 (immune-checkpoint). We report an association between sCD25 levels and both in-hospital and 30-day mortality in COVID-19 patients, together with higher plasma levels compared to CKD patients and healthy controls. Soluble CD25 is generated upon activation, binds to IL-2 and can thus modulate the immune responses [31, 32]. The level has been reported to be higher in severely ill patients [33], higher compared to healthy controls [34] and may reflect the divergence in T cell responses, anti-virus and pro-inflammatory responses [35].

The sPD-L1 level was associated with mortality and was increased in COVID-19 patients compared to both healthy controls and the matched subgroup of CKD patients without COVID-19. Soluble PD-L1 levels are reported to be higher in active SARS-CoV-2 infection [20] and treatment with anti PD-L1 antibodies restores the T cell capacity to reduce the viral load [31]. Soluble PD-L1 is generally regarded as an immune suppressor factor and check point molecule which has been shown to correlate to the higher plasma viral RNA load in COVID-19 patients [32]. Together, current data indicate a potential role for so-called immune checkpoints in the pathogenesis of COVID-19 and thus also as a potential treatment target [36].

Interestingly, we observed significant correlations between sPD-L1 and sCD25 as well as between sCD95 and sCD25. These associations support a present operational imbalance between T cell suppression and activation in COVID-19 patients. Yet, the interpretation should be made with caution due to the complexity of the immune response network with several redundances and compartment related functions.

Our study has limitations. We have included a relatively small patient sample size and we lack exact knowledge about the cause for the increase in plasma creatinine at the time of sampling. This may be due to SARS-CoV-2 infection with AKI, preexisting CKD, dehydration due to fever/gastrointestinal symptoms or acute hemodynamic instability. We have no information about a possible co-infection or superinfection with bacteria which may affect the immune response in patients with SARS-CoV-2 infection. Moreover, we could not adjust for all three parameters age, sex, eGFR at once for analysis of the mortality risk due to low number of events. Covid-19 is a disease with an individual course, and we cannot state at which exact stage of disease the samples were drawn. This should be kept in mind since the time point for inclusion was based on the clinical need for hospitalization, and not on laboratory parameters. Our study has several strengths, such as an early enrollment and blood sampling of patients at time of admission with systematic recording of patient information, level of care and treatments. Furthermore, this study was performed on treatment-naïve patients who were hospitalized early during the first pandemic wave when there were no established treatment recommendations (e.g. anticoagulants and high doses steroids) available. This is important when studying immune system features. Moreover, we compared a subgroup of patients with COVID-19 to sex- and eGFR-matched controls, which has not been reported earlier.

## Conclusion

In conclusion, we confirm a significant impact of impaired kidney function on mortality in patients with COVID-19 and add new important information on the influence of increased levels of BAFF, sCD25 and sPD-L1 on the mortality risk. Our data warrants additional analysis to identify new prognostic and therapeutic approaches.

## Supplementary Information

**Additional file 1. **

## Data Availability

All data generated or analyzed during this study are included in this published article and its supplementary information files.

## Abbreviations

AKI: Acute kidney injury
BAFF: B cell activating factors
CD: Soluble Cluster of differentiation
CKD: Chronic kidney disease
CKD-EPI: Chronic Kidney Disease Epidemiology Collaboration
COVID: Coronavirus disease
CRP: C-reactive protein
EDTA: Ethylenediaminetetraacetic acid
eGFR: Estimated glomerular filtration rate
IFN: Interferon
IL: Interleukin
IQR: Interquartile range
NLR: Neutrophil-to-lymphocyte ratio
RT-PCR: Reverse‐transcriptase polymerase chain reaction
SARS-CoV-2: Severe Acute Respiratory Syndrome Coronavirus-2
sPD-L: Soluble Programmed death-ligand
WBC: White blood cell
