# Supplementary material for: “Role of kidney function and concentrations of BAFF, sPD-L1 and sCD25 on mortality in hospitalized patients with COVID-19”
Source: BMC Nephrol. 2022 Sep 2;23:299. doi: 10.1186/s12882-022-02924-2 (PMC9438228; doi:10.1186/s12882-022-02924-2)
Supplement: Supplementary file 1 — Additional file 1. [file 12882_2022_2924_MOESM1_ESM.docx]

**Supplementary- Figure and Table**

**
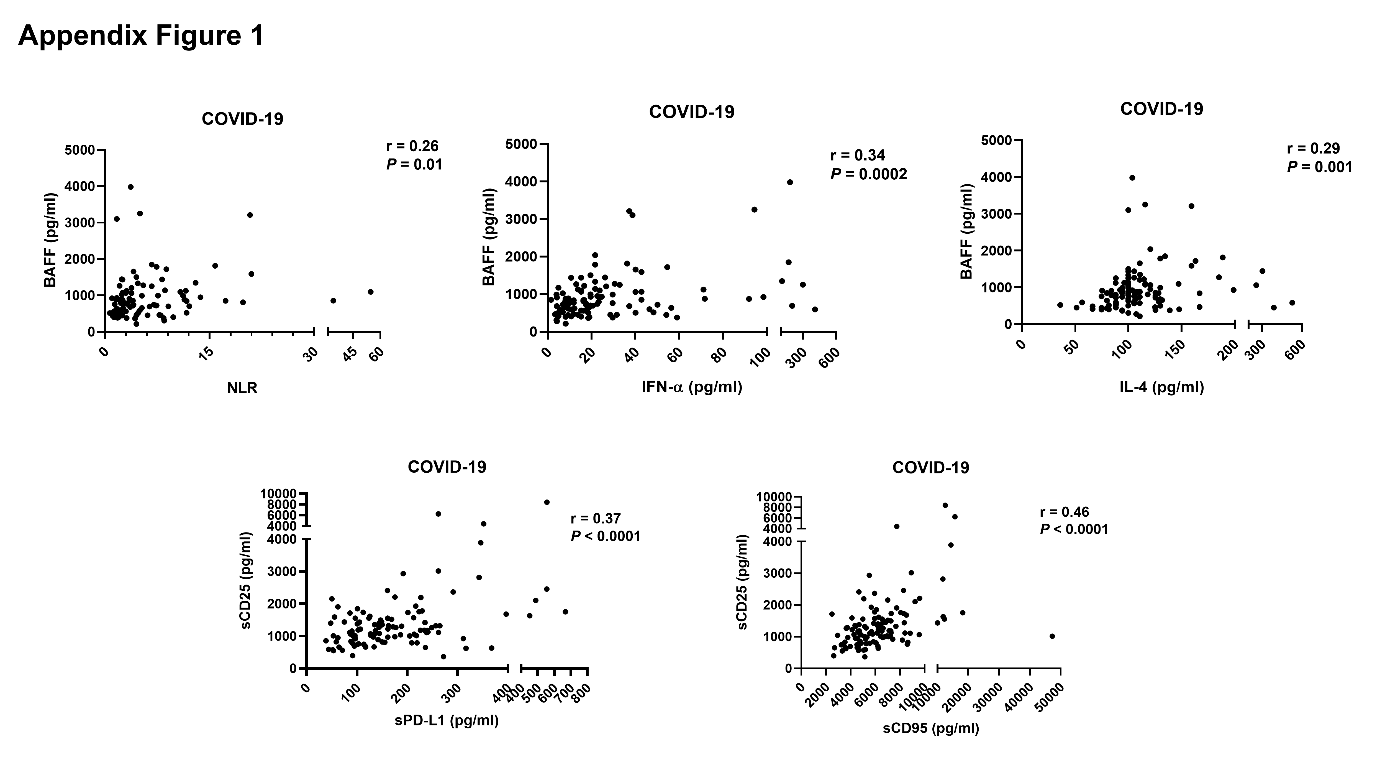
**

Scatter plots presenting the relationship between neutrophil-to-lymphocyte ratio and BAFF, IFN-α and BAFF, IL-4 and BAFF, sCD25 and sPD-L1 as well as sCD25 and sCD95. Spearman´s rank correlation test with 95% confidence interval (CI) was applied.

**Appendix Table 1.** Comparison between COVID-19 patients with impaired kidney function (eGFR≤ 84 ml/min/1.73m^2^) and sex and eGFR-matched patients with chronic kidney disease (CKD). The comparison between groups was analyzed by nonparametric Mann-Whitney U-test and a P < 0.05 was considered a significant difference.

|  | COVID-19 (N=29) | | CKD patients (N=29) | | *P (K-W)* |
| --- | --- | --- | --- | --- | --- |
|  | Median | IQR 25-75% | Median | IQR 25-75% |  |
| Age (years) | 68 | 61-81 | 56 | 49-58 | **0.000** |
| eGFR^2^ (ml/min/1.73m^2^) | 50 | 29.5-62.7 | 51 | 31-64 | NS |
| *B cell modulation* | | | | |  |
| BAFF^4^ (pg/ml) | 929.5 | 706.5-1265 | 450.5 | 405-551 | **0.000** |
| IL-4^5^ (pg/ml) | 125 | 100-153 | 133.5 | 111-208 | NS |
| IFN-α^6^ (pg/ml) | 22 | 15-37 | 9 | 4.5-19 | **0.01** |
| *T cell modulation* | | | | |  |
| sCD25^7^/IL-2Rα^8^ (pg/ml) | 1555 | 1143-2158 | 767 | 582-854 | **0.000** |
| sCD40L (pg/ml) | 1474 | 1238-1726 | 7663 | 5771-9955 | **0.000** |
| sPD-L1^9^ (pg/ml) | 230 | 141-344.5 | 85 | 64-109 | **0.000** |
| sCD95/Fas (pg/ml) | 7273 | 5914-10886 | 8911 | 7814-10496 | NS |

^1^ Body mass index,^2^ Estimated glomerular filtration rate, ^3^ C-reactive protein, ^4^ [B-cell activating factor,](https://en.wikipedia.org/wiki/B-cell_activating_factor) ^[5](https://en.wikipedia.org/wiki/B-cell_activating_factor)^ [Interleukin 4,](https://en.wikipedia.org/wiki/B-cell_activating_factor) ^[6](https://en.wikipedia.org/wiki/B-cell_activating_factor)^ [Interferon-α,](https://en.wikipedia.org/wiki/B-cell_activating_factor) ^[7](https://en.wikipedia.org/wiki/B-cell_activating_factor)^ [Soluble cluster of differentiation 25,](https://en.wikipedia.org/wiki/B-cell_activating_factor) ^[8](https://en.wikipedia.org/wiki/B-cell_activating_factor)^ [Interleukin 2 receptor- α,](https://en.wikipedia.org/wiki/B-cell_activating_factor)^[9](https://en.wikipedia.org/wiki/B-cell_activating_factor)^ [Soluble programmed death-ligand 1](https://en.wikipedia.org/wiki/B-cell_activating_factor)
